# Enhanced Biocompatibility in Anodic TaO_*x*_ Nanotube Arrays

**DOI:** 10.1186/s11671-017-2325-0

**Published:** 2017-10-03

**Authors:** Yu-Jin Zeng, Sheng-Chen Twan, Kuan-Wen Wang, Her-Hsiung Huang, Yen-Bin Hsu, Chien-Ying Wang, Ming-Ying Lan, Sheng-Wei Lee

**Affiliations:** 1 Institute of Materials Science and Engineering, National Central University, Taoyuan, 320 Taiwan Republic of China; 2 Department of Dentistry, National Yang-Ming University, Taipei, 11221 Taiwan Republic of China; 3 Department of Otolaryngology Head and Neck Surgery, Taipei Veterans General Hospital, Taipei, 112 Taiwan Republic of China; 4 School of Medicine, National Yang-Ming University, Taipei, 112 Taiwan Republic of China; 5 Department of Emergency, Taipei Veterans General Hospital, Taipei, 112 Taiwan Republic of China

**Keywords:** Biocompatibility, TaO_*x*_ nanotubes, Anodic oxidation, Hydrophilicity, Human fibroblast cells

## Abstract

This study first investigates the biocompatibility of self-organized TaO_*x*_ nanotube arrays with different nanotube diameters fabricated by electrochemical anodization. All as-anodized TaO_*x*_ nanotubes were identified to be an amorphous phase. The transition in surface wettability with TaO_*x*_ nanotube diameters can be explained based on Wenzel’s model in terms of geometric roughness. In vitro biocompatibility evaluation further indicates that fibroblast cells exhibit an obvious wettability-dependent behavior on the TaO_*x*_ nanotubes. The 35-nm-diameter TaO_*x*_ nanotube arrays reveal the highest biocompatibility among all samples. This enhancement could be attributed to highly dense focal points provided by TaO_*x*_ nanotubes due to higher surface hydrophilicity. This work demonstrates that the biocompatibility in Ta can be improved by forming TaO_*x*_ nanotube arrays on the surface with appropriate nanotube diameter and geometric roughness.

## Background

Tantalum (Ta) is a rare, hard, highly corrosion-resistant, and bioinert metal [1–3]. Oxidation of the tantalum material, by forming a very thin, impenetrable oxide film on its surface, contributes to its biocompatibility. The high flexibility and biocompatibility of tantalum render its clinical applications, such as dental implants, orthopedic implants, and bone reconstruction [4–6]. Recently, tantalum was found to have better biocompatibility than titanium, such as more abundant extracellular matrix formation, excellent cellular adherence and growth, and a much higher living cell density on the surface [7–9]. On the other hand, several studies have proved that the distinctive physico-chemical property of nanostructured surface geometry is the main factor influencing cell behavior [10–12]. The ideal biomaterial surface should be able to provide the optimal environment for cell ingrowth. Ruckh et al. demonstrated that anodized Ta nanotubes provide a substrate for enhanced osseointegration when compared to flat surface [13]. A recently developed porous tantalum material, mimicking the properties of bone, allows the soft tissue and bone ingrowth which provides good biological fixation [14–17]. The high stability and healing potential of porous tantalum help to fuse the gaps between bone structures during reconstructive surgery. The porous tantalum thus regained much interest in the biomaterial field due to its several advantages compared to other grafts, such as no donorsite morbidity, high stability, excellent osseointegrative properties, and prevention of potential risk of transmission of infectious diseases [18–21]. A recent clinical review showed that patients who received porous tantalum acetabular cups had a higher degree of implant fixation compared to those with hydroxyapatite-coated titanium (Ti) cups [22–25].

Recently, we have developed self-organized TiO_2_ nanotubes with different diameters by utilizing an electrochemical anodization method [26, 27]. We found that human fibroblast cells show more obvious diameter-specific behavior on the supercritical CO_2_ (ScCO_2_)-treated nanotubes than those on the as-anodized ones [27]. We further fabricated Ag-decorated TiO_2_ nanotubes by the electron-beam evaporation method and found the smallest diameter (25-nm-diameter) Ag-decorated nanotubes exhibited the most obvious biological activity in promoting adhesion and proliferation of human fibroblasts and also human nasal epithelial cells [26]. In this study, we fabricated TaO_*x*_ nanotubes with different diameters by the similar electrochemical anodization method. The cell behavior, including cell adhesion, and proliferation, in response to the diameter of TaO_*x*_ nanotubes were investigated. The objective of this research is to study the biocompatibility of self-organized TaO_*x*_ nanotube arrays with different nanotube diameters fabricated by electrochemical anodization.

## Methods

### Preparation of TaO_*x*_ Nanotubes

Ta sheets were purchased from ECHO Chemical (thickness of 0.127 mm, 99.7% purity, CAS No. 7440-25-7). Before the anodization process, Ta sheets were ultrasonically cleaned in acetone, isopropanol, ethanol, and water. All anodization experiments were performed at 20 °C in sulfuric acid solution containing 4.9 wt% HF, which was prepared from reagent-grade chemicals and deionized water. A two-electrode electrochemical cell with Ta as the anode and Pt as the counter electrode was employed. The voltages were adjusted from 10 to 40 V to result in TaO_*x*_ nanotube diameters ranging from 20 up to 90 nm. Low-intensity UV-light irradiation (about 2 mW/cm^2^) with fluorescent black-light bulbs on TaO_*x*_ nanotube samples for 8 h was done before the biocompatible tests.

### Material Characterization

The surface morphology, inner and outer diameter, wall thickness, and length of TaO_*x*_ nanotubes were characterized by scanning electron microscopy (SEM). X-ray diffraction (XRD) and transmission electron microscopy (TEM) equipped with an energy dispersion spectrometer (EDS) were employed to examine the crystalline structure of the TaO_*x*_ nanotube arrays. Contact angle measurements were carried out to evaluate the surface wettability of the TaO_*x*_ nanotube samples by the extension method using a horizontal microscope with protractor eyepiece. Water and culture medium were used as test liquids for the measurements.

### Human Fibroblast Cell Culture

MRC-5 human fibroblasts (BRCC, Bioresource Collection and Research Center, Hsinchu, Taiwan, BCRC No. 60023) were plated in 10-cm tissue culture plate and cultured with Eagle’s minimum essential medium (Gibco) containing 10% fetal bovine serum (FBS), 2 mM l-glutamine, 1.5 g/L sodium bicarbonate, 0.1 mM non-essential amino acids, and 1.0 mM sodium pyruvate and in 5% CO_2_ at 37 °C. Cells were then seeded on to the autoclaved TaO_*x*_ sheets placed on the bottom of 12-well culture plate (Falcon) for further study.

### Cell Adhesion Assay

Cells were seeded on each TaO_*x*_ sheet at a density of 2.5 × 10^3^ cells/cm^2^ and incubated in 5% CO_2_ at 37 °C for 3 days and rinsed twice with PBS. The adherent cells on the substrate were fixed for 1 h in 4% paraformaldehyde at room temperature, followed by two washes in phosphate buffered saline (PBS) and permeabilization with 0.1% Triton X-100(Sigma-Aldrich) in PBS for 15 min at 4 °C. After washing with PBS, the actin filament was labeled by incubating with rhodamine phalloidin (Life Technologies) at room temperature for 15 min. Then cell nuclei were stained by incubating with diamidino-2-phenylindole (DAPI) (Thermo FisherScientific) for 5 min. Cells were analyzed under a fluorescent microscope (AX80, Olympus) to examine the cell adhesion morphology and cytoskeletal arrangement. For SEM observation, cells were fixed with 2.5% glutaraldehyde solution (Merck) for 1 h at room temperature, then rinsed in PBS solution twice, dehydrated in a series of ethanol (40, 50, 60, 70, 80, 90, and 100%) and critical point dried with a critical-point dryer (CPD 030, Leica). A thin platinum film was coated on the samples before SEM observation.

### Cell Proliferation Assay

Cells were seeded on each TO_*x*_ substrates at density of 1 × 10^4^ cells/cm^2^ and cultured for 1 week. After 1 week, the samples were rinsed with PBS twice and the cell proliferation was estimated by using WST-1 reagent kit (Roche, Penzberg, Germany). The medium containing 10% WST-1 cell proliferation reagent was added to each specimen and incubated in humidified atmosphere of 5% CO_2_ at 37 °C for 2 h. The solution of each well were transferred to a 96-well plate. The absorbance of solution was measured at 450 nm using the spectrophotometer (Spectral Max250).

### Statistical Analysis

All experiments were carried out in triplicate, and at least three independent experiments were performed. Data were presented as mean ± standard deviation (SD) and analyzed by analysis of variances (ANOVA) using SPSS 12.0 software (SPSS Inc.). A *p* value of < 0.05 was considered statistically significant.

## Results and Discussion

**Fig. 1:**
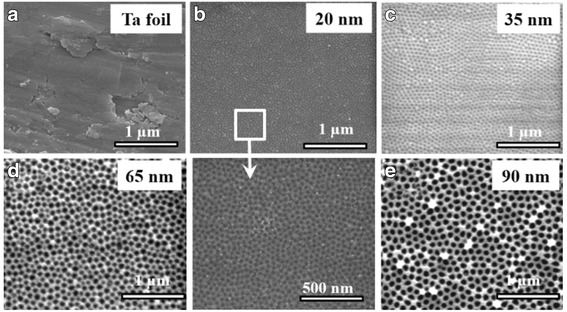
SEM images showing the **a** Ta foil surface and self-organized TaO_*x*_ nanotubes with diameters of **b** 20, **c** 35, **d** 65, and **e** 90 nm, respectively

**Fig. 2:**
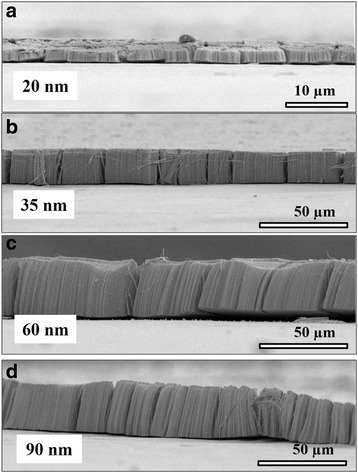
SEM images showing the cross-sections of TaO_*x*_ nanotubes with diameters of **a** 20, **b** 35, **c** 65, and **d** 90 nm, respectively

**Fig. 3:**
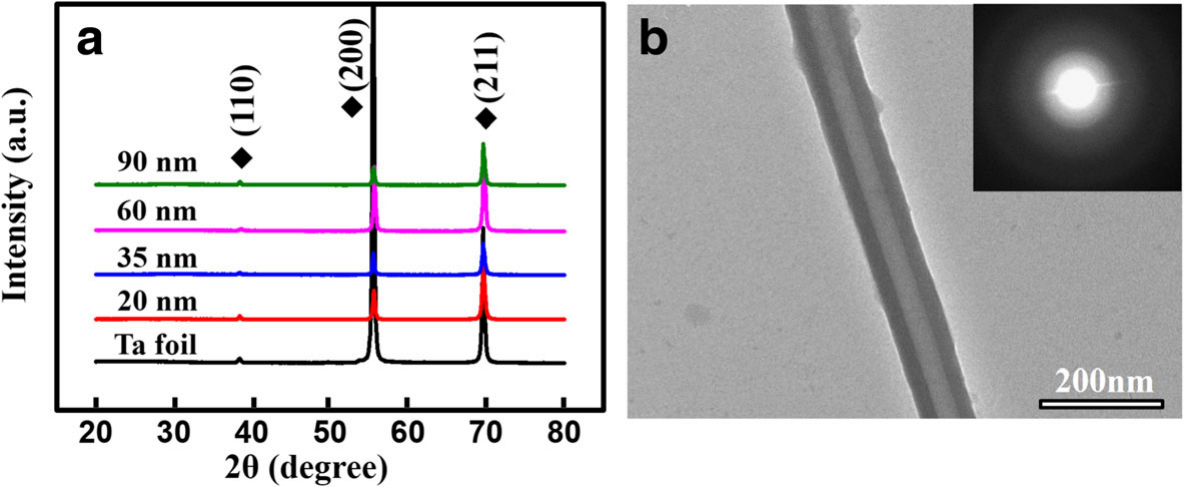
**a** XRD spectra of as-anodized TaO_*x*_ nanotubes of different diameters and **b** TEM image taken from an as-anodized TaO_*x*_ nanotube with the diameter of 90 nm. The inset also shows the corresponding diffraction pattern

Figure 1a–e shows the SEM images of the flat Ta foil and as-anodized TaO_*x*_ nanotube arrays with an average nanotube diameter of 20, 35, 65, and 90 nm, respectively. All as-anodized TaO_*x*_ nanotubes exhibit well-defined nanotubular structure, and their nanotube diameters were nearly proportional to the applied voltages. Among these samples, the 20-nm-diameter nanotubes show relatively unclear nanotubular surface, as shown in the enlarged area taken from Fig. 1b. This observation can be attributed to the weaker field strength under low-voltage operation in the anodization process. Figure 2 further shows the cross-session of all TaO_*x*_ nanotubes and their corresponding nanotubular lengths. XRD and TEM analyses were employed to further identify the TaO_*x*_ nanotube crystallinity. As shown in the XRD spectra of Fig. 3a, only peaks related to the Ta foil are observed (JCPDS Card no. 04–0788), suggesting that as-anodized TaO_*x*_ nanotubes are possibly amorphous phase. Figure 3b shows a representative TEM image taken from a 90-nm-diameter TaO_*x*_ nanotube peeled off from the as-anodized sample, revealing a well-defined nanotubular structure. The spotless diffraction pattern in the inset confirms that the TaO_*x*_ nanotubes are non-crystalline.

**Fig. 4:**
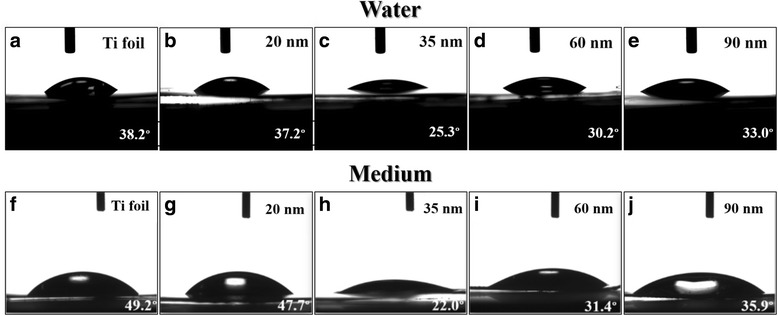
**a**–**j** Optical images showing water and culture medium droplets on the **a**,**f** Ta foil surface and self-organized TaO_x_ nanotubes with diameters of **b**,**g** 20, **c**,**h** 35, **d,i** 65, and **e**,**j** 90 nm, respectively. Contact angles are denoted in the images

**Fig. 5:**
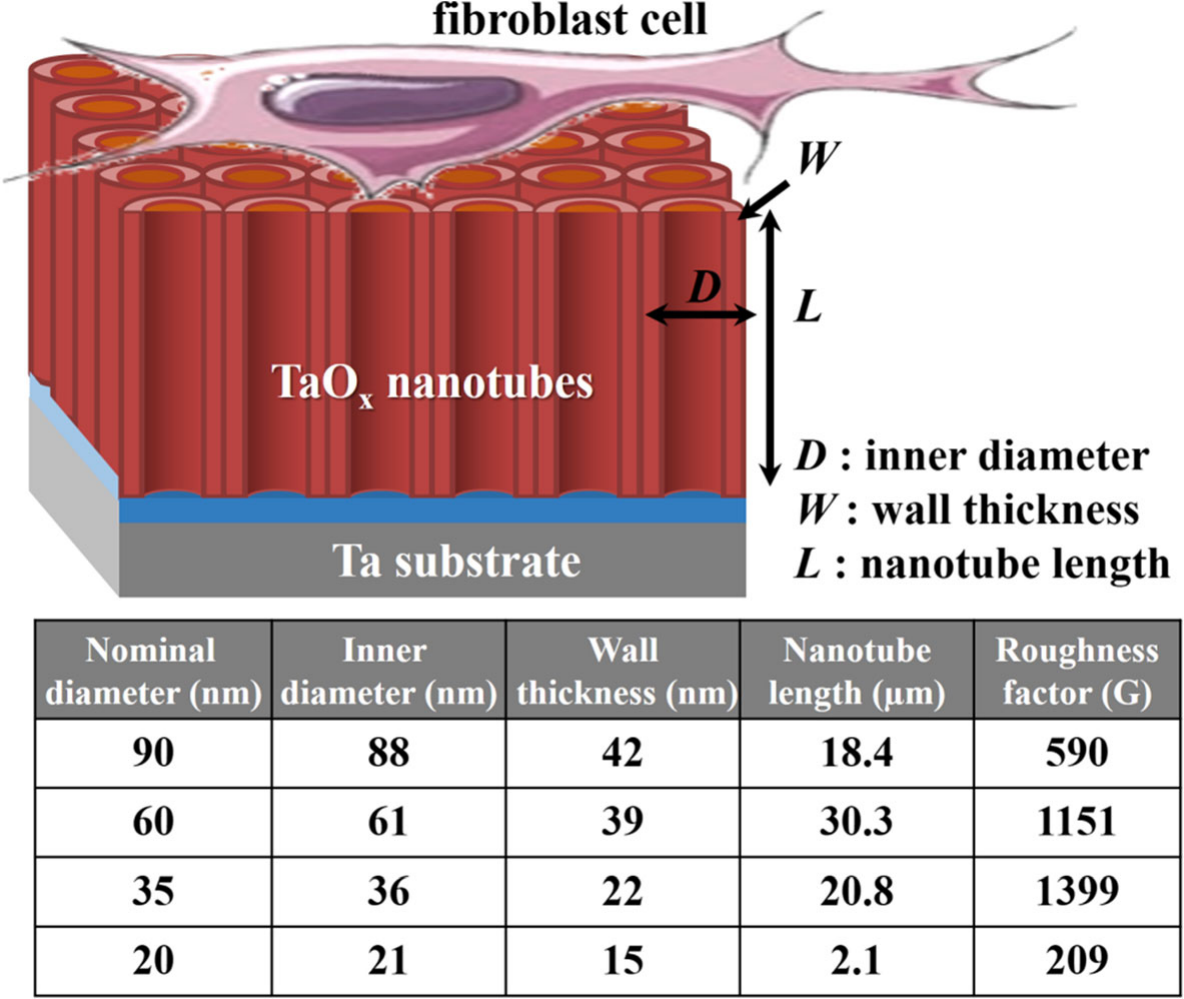
Schematic diagram of an idealized nanotubular structure with inner diameter *D*, wall thickness *W*, and nanotube length *L*. The calculated roughness factors for all nanotube samples in this study are summarized in the table

The previous study has reported that cell attachment, spreading, and cytoskeletal organization are significantly better on hydrophilic surfaces relative to hydrophobic surfaces [28]. Das et al. further indicated that a low contact angle implies high surface energy, which is also a crucial factor that contributes to better cell attachment [29]. It is thus essential to understand the influence of TaO_*x*_ nanotube topography on the surface wettability. As shown in Fig. 4, all as-anodized TaO_*x*_ nanotubes are highly hydrophilic since their contact angles are much smaller than 90°. Furthermore, their contact angles were found to monotonically decrease with decreasing nanotube diameter to 35 nm and then inversely increase as the diameter decreases to 20 nm. We also find that the TaO_*x*_ nanotube samples show the similar trend when using either water or culture medium as testing liquids. We attempt to explain the observed wettability behavior based on Wenzel’s law, which describes the small contact angle on hydrophilic materials [30]. In Wenzel’s model, an increase of surface roughness in hydrophilic material will result in a smaller contact angle, and water will fill the grooves below the droplet. Here, we use the roughness factor, i.e., the physical surface area of nanotubes per unit of projected area, to evaluate the geometric roughness of TaO_*x*_ nanotube samples [31]. As shown in Fig. 5, with inner diameter *D*, wall thickness *W*, and nanotube length *L*, the purely geometric roughness factor *G* can be calculated as [4π*L*{*D* + *W*}*/*{√3*(D* + 2 *W)* ^2^}] + 1*.* This calculation assumes all surfaces of the nanotubes to be perfectly smooth. The calculated roughness factors for all nanotube samples are summarized in the table of Fig. 5. Except the 20-nm-diameter sample, the smaller diameter nanotubes have the larger geometric roughness and thus are thought to exhibit better hydrophilicity according to Wenzel’s model. This inference is consistent with our result that the contact angle decreases with decreasing nanotube diameter to 35 nm. It also well explains that the 20-nm-diameter nanotubes that exhibit relatively unclear nanotubular surface show smaller geometric roughness and poorer hydrophilicity than other ones.

**Fig. 6:**
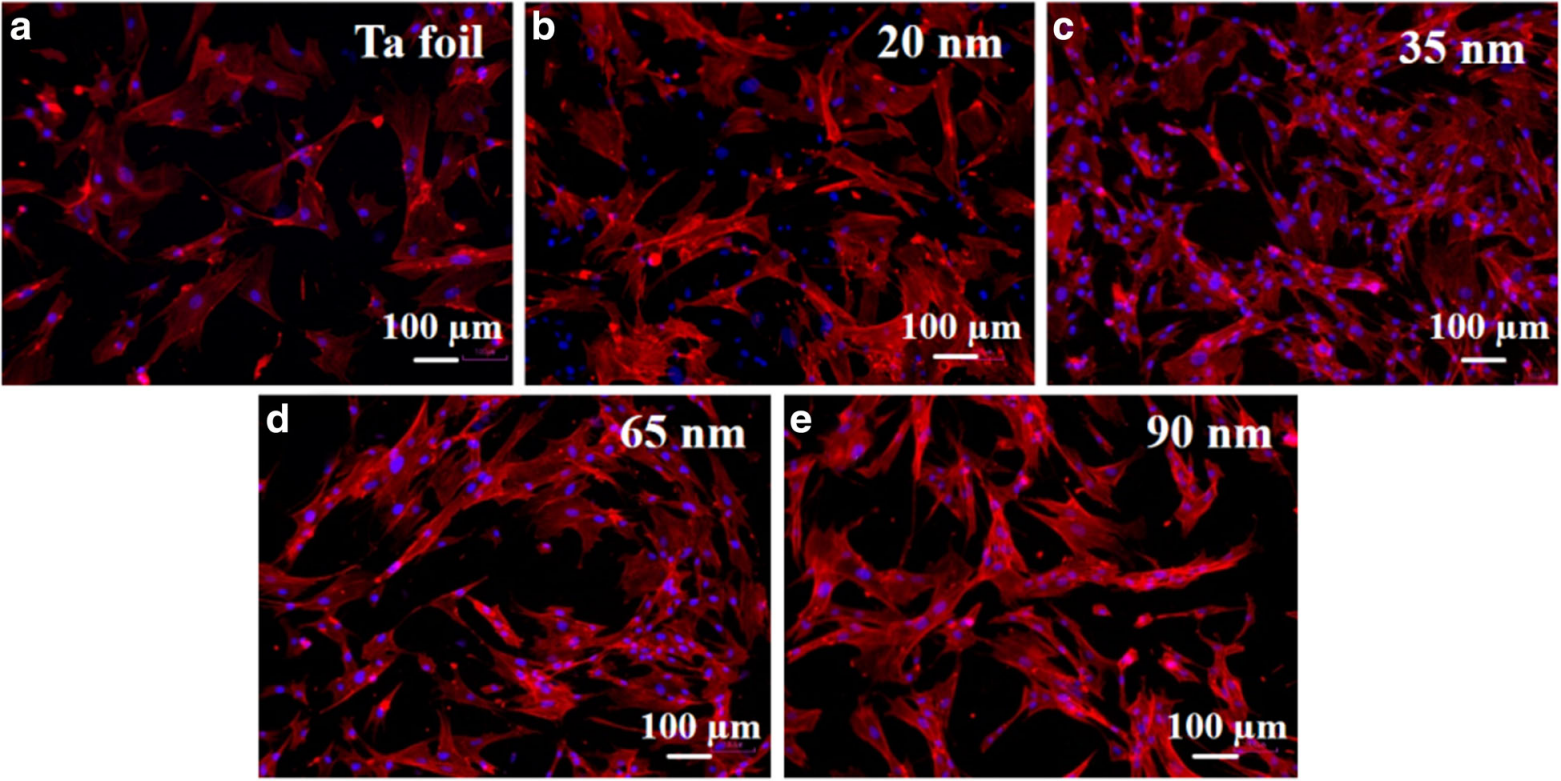
Fluorescence microscopy images of the fibroblast cell attachment on the **a** Ta foil and self-organized TaO_*x*_ nanotubes with diameters of **b** 20, **c** 35, **d** 65, and **e** 90 nm, respectively. The red fluorescence indicates cytoskeletal protein actin filament, and the blue fluorescence indicates nuclei

**Fig. 7:**
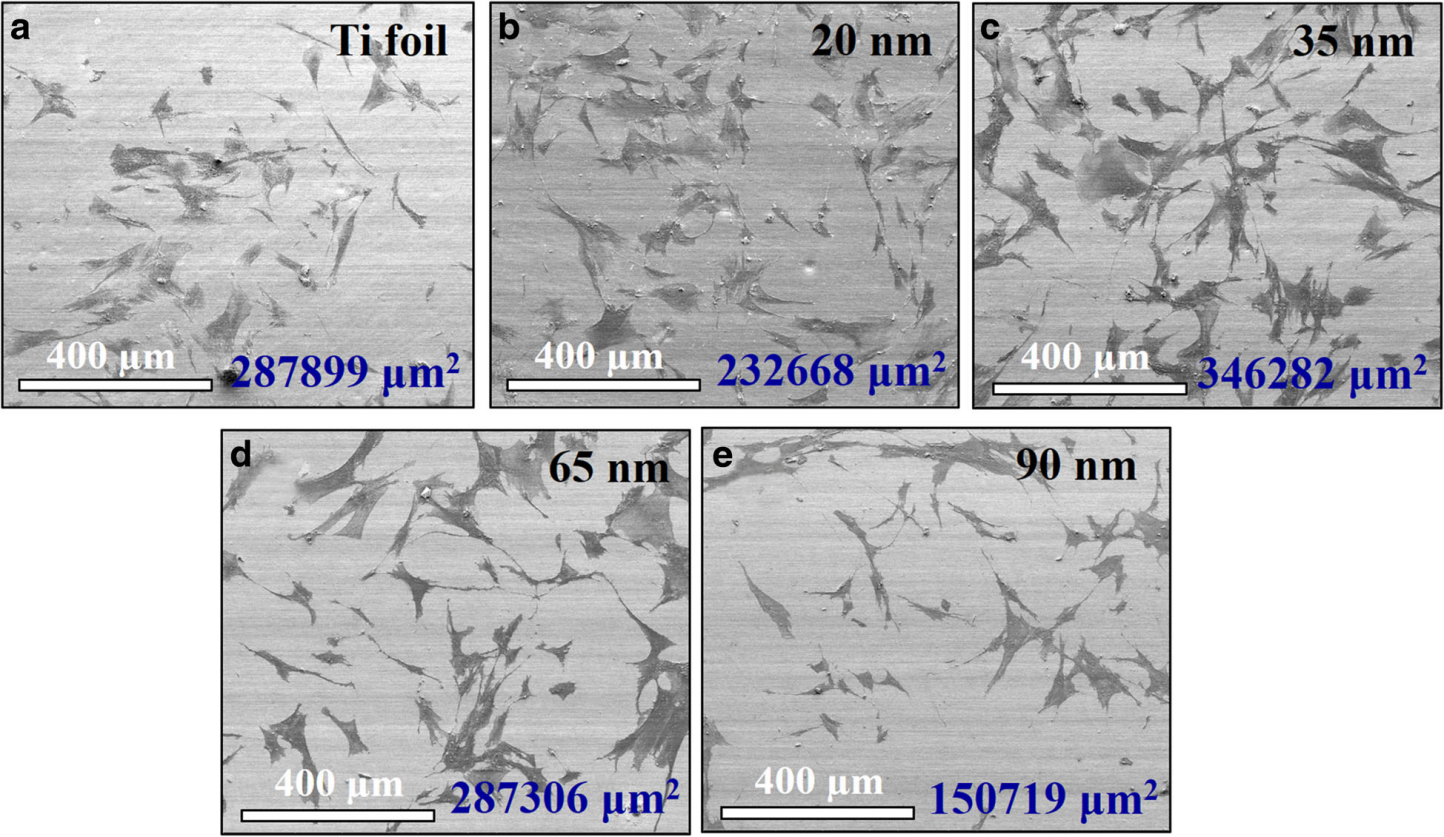
**a**–**e** SEM images showing the cell adhesion and proliferation of human fibroblast cells on the **a** Ta foil surface and self-organized TaO_x_ nanotubes with diameters of **b** 20, **c** 35, **d** 65, and **e** 90 nm, respectively. The coverage areas of cells on the samples estimated by ImageJ software are denoted in the images

The human fibroblast cell behavior in response to the flat Ta foil and TaO_*x*_ nanotube arrays was further studied. To evaluate the fibroblast cell attachment on the TaO_*x*_ nanotubes, cytoskeleton actin was stained with rhodamine phalloidin to express red fluorescence and nuclei stained with DAPI to express blue fluorescence. The actin immunostaining shows distinguishable cell-material contact morphology for the flat Ta foil and TaO_*x*_ nanotubes of different diameters (see Fig. 6). It is well known that cells have to adhere on material surface first and then spread for further cell division. Better cell adhesion can cause more activation of intracellular signaling cascades through integrin coupled to actin cytoskeleton [32–34]. FE-SEM was used for the detailed observation of cell adhesion (see Fig. 7). The fibroblasts on the 35-nm-diameter reveal excellent cell adhesion with an elongated flatten morphology. On the other hand, those fibroblasts on the Ta foil and 90-nm-diameter TaO_*x*_ nanotubes show less attached cells and lack of cell spreading to some extent. The coverage area of cells on the nanotubes was further estimated by using ImageJ software and noted in these SEM images. Similar to the trend of contact angles, the coverage area was found to monotonically decrease with decreasing nanotube diameter to 35 nm and then inversely increase as the diameter decreases to 20 nm. The 35-nm-diameter TaO_*x*_ nanotube indeed shows the largest cell coverage area. It is known that cells recognize surface features when a suitable site for adhesion has been detected. It is supposed that cells can stable their contacts onto the TaO_*x*_ nanotubes by forming focal adhesions and mature actin fibers, followed by recruiting tubulin microtubules [35]. The actin cytoskeleton is linked to integrins which are located within the adhesions. Our findings suggest that the cytoskeleton on the 35-nm-diameter nanotubes could be formed better than those on the flat Ta foil or other TaO_*x*_ nanotube arrays.

**Fig. 8:**
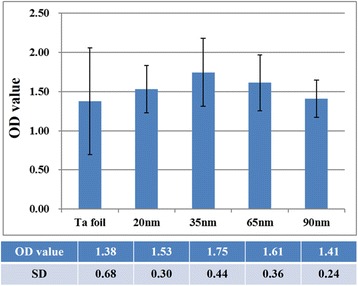
Optical densities (QD) measured after the culture of human fibroblast cells on the Ta foil and self-organized TaO_*x*_ nanotubes of different diameters. The OD values with their standard deviations are listed as an attached table

The WST-1 assay was employed to further evaluate the fibroblast cell proliferation on the TaO_*x*_ nanotubes with different diameters. Figure 8 shows the comparison of optical densities measured from the WST-1 assay results. We find that cell proliferation is highest for the 35-nm-diameter TaO_*x*_ nanotube sample. However, there is no significantly difference between Ta group and TaO_*x*_ nanotube arrays. In addition, the cell proliferation and surface wettability exhibit a nearly similar trend with the TaO_*x*_ nanotube diameters. This observation suggests that not only nanotube diameter but also surface wettability strongly influences the cell adhesion and the following spreading. In other words, compared to the 35-nm-diameter nanotubes, the 20-nm-diameter ones may give more focal points for fibroblast cells, but its poorer hydrophilicity eliminates some effective focal contacts and thus impedes the cell attachment. Eventually, the 35-nm-diameter TaO_*x*_ nanotubes reveal the highest biocompatibility among all samples.

## Conclusions

In conclusion, this work studies the biocompatibility of anodized TaO_*x*_ nanotubes with different nanotube diameters. All anodized TaO_*x*_ nanotubes were identified to be mainly amorphous phase. We discuss the transition in surface wettability with TaO_*x*_ nanotube diameters based on Wenzel’s model. In vitro biocompatibility evaluation further indicates that fibroblast cells exhibit an obvious wettability-dependent behavior on the TaO_*x*_ nanotube arrays. The 35-nm-diameter TaO_*x*_ nanotube arrays reveal the best biocompatibility among all nanotube samples. This enhancement could be attributed to highly dense focal points provided by TaO_*x*_ nanotubes due to higher surface hydrophilicity. This study demonstrates that the biocompatibility in Ta can be improved by forming TaO_*x*_ nanotube arrays with appropriate nanotube diameter and geometric roughness.
